# Patient experience with eosinophilic esophagitis symptoms and impacts on daily life based on in-trial qualitative interviews

**DOI:** 10.1186/s41687-025-00836-x

**Published:** 2025-01-08

**Authors:** Calvin N. Ho, Harald Fjällbrant, Evan S. Dellon, Cecilio Santander, Marc E. Rothenberg, Julie Bailey, Shehan McFadden, Jason Ritchie

**Affiliations:** 1 Patient Centered Science, BioPharmaceuticals Medical, AstraZeneca, Gaithersburg, MD USA; 2 Late-stage Respiratory & Immunology, BioPharmaceuticals R&D, AstraZeneca, Gothenburg, Sweden; 3 Center for Esophageal Diseases and Swallowing, Division of Gastroenterology and Hepatology, University of North Carolina at Chapel Hill School of Medicine, Chapel Hill, NC USA; 4 Department of Gastroenterology and Hepatology, Hospital Universitario de La Princesa, Centro de Investigación Biomédica en Red de Enfermedades Hepáticas y Digestivas (CIBERehd), Instituto de Investigación Sanitaria Princesa (IIS-IP), Universidad Autónoma de Madrid (UAM), Madrid, Spain; 5 Division of Allergy and Immunology, Department of Pediatrics, Cincinnati Children’s Hospital Medical Center, University of Cincinnati College of Medicine, Cincinnati, OH USA; 6 IQVIA, Deerfield, IL USA

**Keywords:** Eosinophilic esophagitis, Benralizumab, Patients’ experiences, Qualitative patient interview, Impact on daily life

## Abstract

**Purpose:**

Eosinophilic esophagitis (EoE), a chronic immune-mediated progressive disease, causes dysphagia, food impaction, abdominal pain, vomiting, and heartburn. EoE requires long-term monitoring and can affect quality of life owing to its symptoms and associated emotional and social burden. This study aimed to understand patients’ experiences with EoE.

**Methods:**

Semi-structured longitudinal interviews were conducted with patients from MESSINA, a phase 3 placebo-controlled trial evaluating benralizumab for EoE. Interviews were held at two different times to assess the impact of EoE on patients’ lives before and during trial participation. Data were analyzed qualitatively to develop detailed patient profiles.

**Results:**

The MESSINA trial was terminated prematurely. Of the 34 patients recruited for the first interview, 15 (44%) completed the second interview and 11 patient profiles were developed. Patients were a demographically diverse group with varying experiences. The primary reported symptom was difficulty swallowing (*n* = 11), leading to serious consequences like choking and hospitalization (*n* = 2). Other symptoms included pain when swallowing (*n* = 7), reflux (*n* = 6), and stomachache (*n* = 6). In the second interview, most (*n* = 9) patients reported moderate improvements in symptoms, while others experienced symptom recurrence or worsening. EoE had a significant negative impact on social and emotional well-being, and professional lives. Trial participation improved emotional well-being for some; however, concerns about the need for ongoing treatment were noted.

**Conclusion:**

This study highlighted emotional and social burdens of EoE. The encouraging feedback on study participation underscores the importance of patient insights in developing holistic management strategies for EoE.

**Supplementary Information:**

The online version contains supplementary material available at 10.1186/s41687-025-00836-x.

## Introduction

Eosinophilic esophagitis (EoE) is a progressive, chronic inflammatory condition of the esophagus characterized by the abnormal presence of eosinophils (a type of white blood cell) in the lining of the esophagus [1, 2]. It has a heterogeneous clinical presentation with various symptoms, including dysphagia, abdominal pain, vomiting, bolus impaction, chest pain, heartburn, and regurgitation [3]. It is the most prevalent cause of chronic esophagitis after gastroesophageal reflux disease (GERD) and the leading cause of dysphagia and food impaction in children and young adults [2, 4]. EoE is a predominantly male disease, with a prevalence at least three times higher in males compared to females [5]. EoE is diagnosed in symptomatic patients who have an eosinophil count of ≥ 15 per high power field (eos/HPF) in an esophageal biopsy [1].

There are limited, though expanding, disease management options for EoE. Non-pharmacologic options focus on removing allergens from the diet and range from self-directed food avoidance to physician- or dietitian-directed regimens. Patients with severe disease who do not respond to these approaches may need to avoid solid food altogether and consume so-called elemental diets (amino acid-based formulas) orally or through a feeding tube. Pharmacologic treatments include proton pump inhibitors and corticosteroids, both of which are being used off-label in many countries. In some regions, dupilumab and/or oral budesonide have received regulatory approval for the treatment of EoE [1]. Research on additional novel treatments is rapidly advancing, with several trials underway to investigate molecules that may restore the esophageal barrier function or target various inflammatory cells or their mediators [1, 6, 7].

EoE is a lifelong disease requiring invasive monitoring with upper endoscopy [6]. Healthcare resource utilization is particularly high for patients with EoE, with contributing factors including diagnostic delays, lack of physician and patient awareness of the disease, frequent healthcare visits during the slow adaptation to symptoms by patients, increased likelihood of emergency department visits, repeated endoscopy under general anesthesia, and costly dietary modifications [8]. EoE symptoms, specifically dysphagia and pain, have a significant impact on health-related quality of life (HRQoL), disrupting and restricting patients’ and care partners’ daily lives [9]. There is considerable emotional burden, as patients with EoE may suffer from mental distress related to the continuous symptoms and risk for acute esophageal food impactions requiring emergency medical intervention [10, 11].

Elimination diets, although effective, can be difficult to sustain, particularly for older children and adults who encounter social situations where sharing food is inevitable (e.g., meals with family, friends, co-workers, or classmates) [3, 12, 13], while the monotony and poor taste associated with such elimination diets contribute to their drawbacks. Furthermore, patients on elemental diets face high costs and social isolation due to the inability to consume solid food [14].

Recent studies have explored patient experiences with EoE and highlighted factors, such as emotional distress, difficulty with feeding, and limitations in social activities, that contribute to poor quality of life [9, 15, 16]. However, there is limited qualitative research that focuses on patient perspectives of living with EoE and on how disease symptoms impact daily lives. Patient insights and perspectives are important to better understand perceptions and quality-of-life impacts, which further allows establishing therapeutic objectives that encompass emotional and social concepts relevant to the burden of EoE.

The aim of this longitudinal qualitative patient interview study was to characterize the patient experience with EoE, focusing on gastrointestinal symptoms, food-related behaviors, and the impact of these symptoms on daily life. The interviews were conducted as a sub-study within an interventional drug trial [17]. The advantage of this longitudinal, in-trial mixed-methods approach was that we could gather information on patient experiences with EoE symptoms both before and during the trial.

## Methods

MESSINA (NCT04543409) was a phase 3 randomized, double-blind, placebo-controlled multicenter clinical trial investigating the use of benralizumab (an injectable monoclonal antibody) for the treatment of EoE. Patients aged between 12 and 65 years with confirmed diagnosis of EoE were included in the trial, which was comprised of three distinct treatment periods: a 24-week double-blind treatment period (Visit 2 [Week 0] to Visit 8 [Week 24]), a 28-week open label treatment period (to Visit 15 [Week 52]), and an open label extension period (to Visit 28 [Week 104]). Patients were required to maintain the same diet during the first year of the trial (up to Visit 15 [Week 52]). However, the trial was terminated prematurely, before patients completed Visit 21. The main results of the MESSINA trial have been published separately [17].

English and Spanish-speaking adult patients who lived in the United Kingdom, United States (US), Canada, or Spain were invited to opt for the qualitative interview study. One-on-one telephone interviews were planned for three time points. The first interview, focused on experiences before the trial and at the beginning of the trial, was conducted after Visit 1 (Weeks − 8 to − 2) and before Visit 3 (Week 4) during the double-blind period. The second interview, focused on experiences during the trial, was conducted after Visit 13 (Week 44) but before Visit 15 (Week 52) during the open label treatment period. The third interview was to be conducted after Visit 21 (Week 76) but before Visit 27 (Week 100) to interrogate experiences since the start of the open label extension period, during which patients were allowed to modify their diet. As the MESSINA trial was terminated prematurely, the third interview was not conducted, therefore this article will focus on the first two interviews.

The interviews were based on semi-structured discussion guides that consisted of open-ended questions about symptoms, the impact of symptoms on HRQoL (first and second interview), and how these impacts might have changed after trial participation (second interview). The first interview lasted 90 min and focused on patient experiences of EoE before diagnosis and before entering the trial. It comprised of four sections, including basic demographic information, EoE symptoms, impacts on daily life, and definition of treatment success.

The second interview also lasted 90 min, and the focus was on patient experiences of EoE symptoms and impacts on daily life, as well as any changes they might have experienced in the months since they entered the trial. In the second interview, apart from the questions included in the first interview, the patients were questioned about meaningful changes in symptoms and symptoms’ impact over the study period.

The interviews were audio-recorded and transcribed, and any patient-identifying data present in the recorded interviews were redacted from the transcripts, and patient names were pseudonymized. Transcripts in Spanish were professionally translated to English prior to analysis.

### Qualitative data analysis

Qualitative data from the interviews were analyzed using both deductive and inductive coding techniques. A pre-defined coding frame was developed and uploaded to a qualitative data analysis software based on the topics included in the interview guides. Codes were also derived from the data as concepts and ideas naturally emerged during coding.

Trained qualitative researchers coded data collected during the patient interviews using a qualitative data analysis software (MaxQDA). The coding process identified and categorized patient concept expressions (patient quotes that exemplify or relate to the reported concepts). Coders reviewed each transcript to identify text that included concept expressions and tag selected text with a code. Coding language and groupings were refined and reconciled between coders as needed.

After coding was complete, data were cleaned to ensure frequency counts and codes for saturation analysis as well as general qualitative coding were accurate, and codes had not been wrongly assigned to text. Once the data were cleaned and any inconsistencies rectified, data were analyzed.

As part of the data analysis, the paired longitudinal interviews from 11 patients were re-analyzed in greater depth and summarized as patient profiles. These patients were selected for profiling based on how well they exemplified the themes that emerged in the research as well as based on their ability to represent both predetermined and emergent categories of patients (e.g., patients from specific countries and treatment groups, or patients with specific types of experiences). This article primarily draws from the 11 patient profiles, though comparisons to the full interview dataset will be noted.

### Integration with clinical trial data

The MESSINA trial was terminated early, after the primary outcomes were assessed at the end of the double-blind period, but before the open-label periods were complete [17]. Some patient-level clinical trial data (e.g., treatment group, baseline characteristics, and patient-reported outcome [PRO] scores) were available to the interview sub-study analysis and interpretation team (including authors CNH, SM, JB, and JR). This team was aware of the finding that there was no meaningful difference between the benralizumab and placebo arms in the primary symptom endpoint of the trial, or in other PRO endpoints. It is worth noting that patient-reported changes are valuable on their own and have been considered, regardless of clinical efficacy outcomes. The lack of efficacy on symptoms and HRQoL at the group level was considered when interpreting individual patient reports of change (or lack of change) in their experience of the disease.

## Results

### Patient demographics

A total of 34 patients were recruited in this qualitative interview study with a mean (standard deviation [SD]) age of 41 (11.6) years at the first interview, ranging from 16 to 67 years (Supplementary Table 1). All patients completed the first interview, and 15 patients completed the second interview.

Among these, 11 patient profiles, with patient names as pseudonyms, were developed based on a demographically representative group presenting with diverse experiences with disease management. Mean age (SD) of these patients during the first interview was 40.6 (13.0) years, ranging from 23 to 62 years, with mostly male patients (*n* = 8/11). The mean time since diagnosis at the first interview was 5.9 years (ranging from less than 1 year to 11 years). Demographic characteristics are shown in Table 1.

**Table 1:** Patient demographics

Demographics	*N* = 11^a^
**Sex**	
Male	8
Female	3
**Age (years)**	
Mean (SD)	40.6 (13.0)
Range	23–62
Not reported	*n* = 1
**Age at diagnosis (years)**	
Mean (SD)	34.2 (12.5)
Range	18–55
Not reported	*n* = 1
**Time since diagnosis ranges**,** n (%)**	
Less than 1 year	1 (9.1%)
1–4 years	4 (36.4%)
5–9 years	3 (27.3%)
10–14 years	3 (27.3%)
15 years or more	0 (0.0%)
Not reported	0 (0.0%)

SD: standard deviation

^a^Of the 11 patients, five were part of the placebo group, and six were part of the treatment group of the MESSINA trial

## Interplay of symptoms and food-related HRQoL

In the first interview, all profiled patients (*n* = 11) reported difficulty swallowing as their primary concern. The challenges with swallowing extended beyond inconvenience, such as choking and food impactions in the esophagus, which resulted in hospitalization for two patients. Moreover, the struggles with swallowing were accompanied by other symptoms, such as pain, nausea, reflux, frequent belching, and persistent coughing. Patients described the sensation of food moving slowly down the esophagus, often causing choking and the need to vomit to clear the throat. Most patients (*n* = 9/11) reported that food bolus impactions were triggered by meat, with other triggers including bread, honey, rice, cake, peanuts, hard vegetables, and fatty foods.

**Fig. 1:**
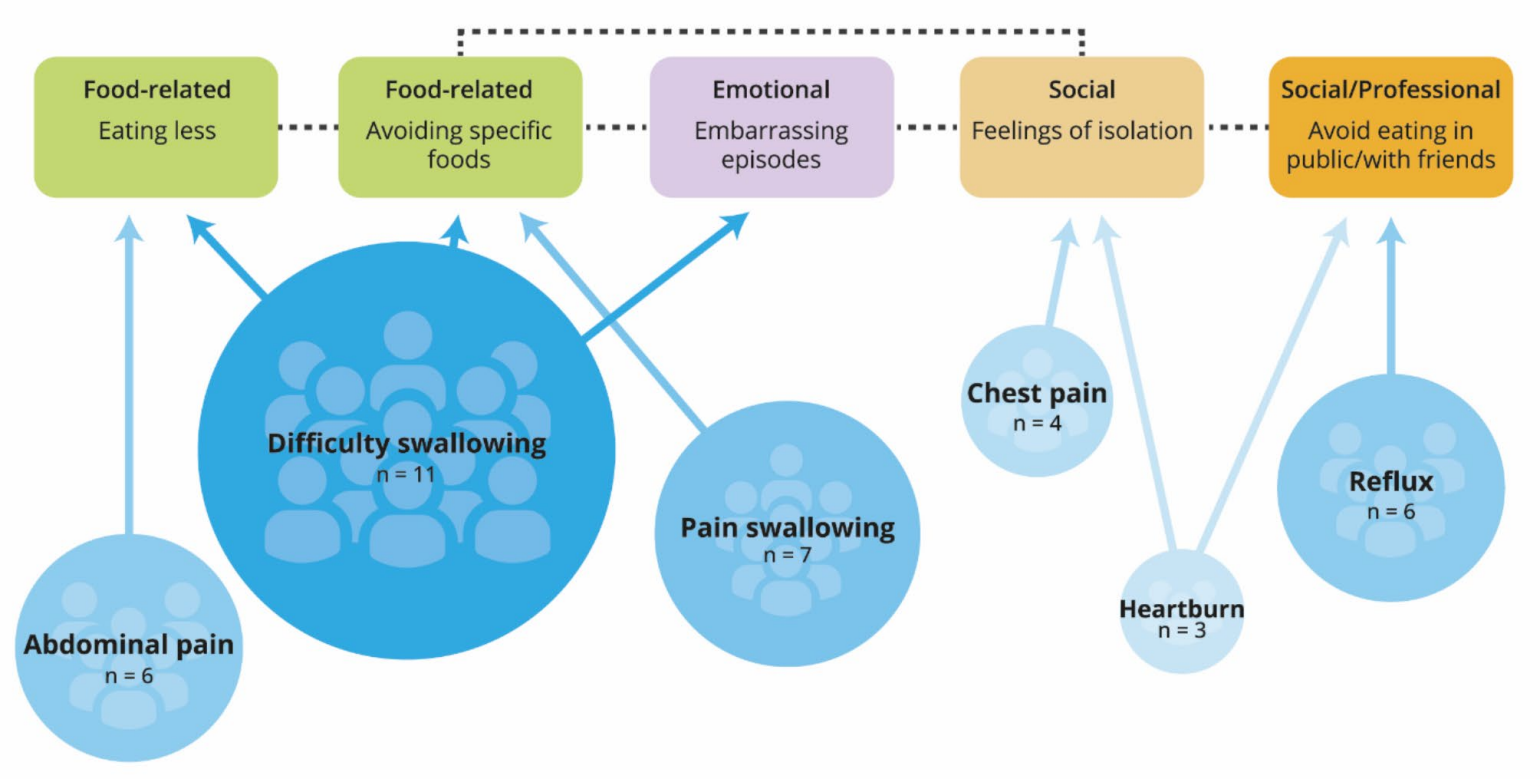
EoE symptoms and impacts on patients’ daily lives

EoE symptoms were closely linked to specific HRQoL effects (Fig. 1). To avoid the embarrassment and panic of choking or having difficulty swallowing, for instance, patients reported eating less food and avoiding trigger foods. Some patients also avoided eating in public, which led to social isolation.

For instance, this excerpt from Patient A’s profile shows how dysphagia is related to multiple symptoms, impacts on HRQoL, and behavioral adaptations. Patient A avoided eating in public out of fear of experiencing an episode, with similar concerns extending to work functions. Patient A’s worry about having an episode in public was so pervasive that they only ate when at home, and consequently felt socially limited.

I don’t really go out to dinner. I don’t even know the last time I was at a restaurant. […] If I was to go somewhere […] it’ll have to be somewhere I’m familiar with. I have to know where the bathroom is at. I need to find a seat that’s close to the bathroom. If the restaurant is really crowded, then I might not even eat because I’m just too anxious and afraid that something might happen. I might have like a bowl of soup or something for dinner.

This worry was not just limited to embarrassment about choking in public. Patient A often worried about the physical and emotional impacts of choking, reporting that they felt fear or panic when food got lodged in his throat. This led to choosing not to eat for extended periods of time, which, in turn, led to occasional fatigue.

I would say ‘yes’ to [experiencing fatigue] and relate it to probably just going long periods of time without food, without calories, and maybe just feeling tired from that. Sometimes I’ll go too long, and I’ll get past that hunger phase and get to the ‘now I just feel sick because I haven’t eaten’ phase.

Similarly, Patient B showed how dysphagia can affect one’s professional life. They noted that, even beyond everyday activities and social life, difficulty swallowing impacted work life, especially because of the expectation to socialize over meals with colleagues.

It affects my work in the sense that I avoid work meals […] Maybe I can be seen as quite an unsociable person possibly because whenever they say, “Let’s go to eat here, we’re going to arrange a meal,” […] but group meals, large ones, I avoid them. […] it can affect me in the sense that people think of me as being a person who doesn’t want to socialize.

Patient B’s work attendance was also affected, reporting sometimes having to miss work following choking episodes.

I had to leave work twice because … but I was already at work. It has happened to me at work. […] But I’ve never missed it saying, ‘I’m not going to work because I’m feeling bad.

Perhaps because of the custom of sharing meals as a common social ritual in her culture, Patient B described limitations in connecting with others because of difficulty swallowing.

Not sharing, for example, if there is a meal with friends and everyone asks for, for example, a steak to share or something like that.[…] I can’t talk, for example, when I’m at a meal, I have to focus on eating, and I can’t have a conversation if I’m not stopping, stopping eating. […] I don’t tend to answer questions and the times I’ve answered it has been horrible because that’s when I’ve had an episode.

By the second interview, most patients (*n* = 9/11) noted moderate improvements in EoE symptoms. Two patients reported no improvement in the symptoms or impacts on daily life. Three patients reported that symptoms related to acid reflux and occasional “spasms” initially improved, but significantly worsened by the time of the second interview. These symptoms emerged as major concerns for these patients, underscoring the unpredictable and variable nature of EoE progression, and they continued to articulate a significant impact of EoE symptoms on their HRQoL in the second interview (Table 2).

**Table 2:** EoE symptoms and impacts on daily life reported during the patient interviews (N = 11)

Symptoms		Impact (social/emotional/professional)		Example quotes from the patient profiles	
First interview, n for number of patients reporting symptom	Second interview, n for number of patients reporting symptom	First interview	Second interview	First interview	Second interview
Difficulty or trouble swallowing n = 11 (all patients)	Difficulty or trouble swallowing n = 10	Social: unpredictable/embarrassing episodes, make frequent trips to bathroom, avoid eating with friends in public Emotional: feelings of frustration, embarrassment, anxiety, and isolation Food-related impacts: avoiding specific foods, eating less, and burden of having to order special foods	Social: More engaged socially; greater interest in attending events Emotional: Increase in “confidence” and improved emotional outlook Work-related impacts were still prevalent	*“It’s mainly trouble with swallowing and impaction. I have food consistently stick*,* I would say*,* a couple of inches above my sternum. And there are some cases where I’ve never been able to chew*,* gag*,* or cough it back up*,* and I’ve never been able to swallow it. So*,* I have had*,* I think*,* at least I had close to 18 boluses in the last ten years that had to be surgically removed.”*	*“[Difficulty swallowing]*,* in particular*,* that happens less and less. Sometimes*,* on the odd occasion*,* it happens. But not even every week. It happens less often than that. [The choking] is getting less and less*,* but yes. In the past*,* [it happened] a lot more than now.”*
Discomfort or pain when swallowing n = 7	Discomfort or pain when swallowing n = 6		No changes reported	*“I eat mainly one meal a day. And I’ve done that because of the pain involved with eating*,* or the discomfort involved with eating*,* or the headache that eating can—I mean*,* it sucks*,* but that’s the kind of diet I put myself on. Everybody loves to fast*,* right?”*	*“I would have to say that [the tingling] still is—that still is the issue that I would—I can feel when it’s going to happen because then I know just to stop*,* not just go about with the meal or anything*,* just to give it time. It happens less frequently*,* and the resulting pain is less*,* but yeah*,* I always have the—it doesn’t take me by surprise. I always can feel that it’s coming.”*
Reflux n = 6	Reflux n = 3	Food-related impacts: avoiding specific foods, eating less, and avoiding eating in public Emotional impact: feeling anxious or worried	Feeling less embarrassed about choking in public	*“You start having a lot of very thick saliva*,* and that’s when you get nauseous*,* and there have been times like I suddenly feel like I’m going to throw up*,* but nothing comes up … It’s stuck down there*,* the food doesn’t go down*,* and if you don’t throw it up*,* you like choke. It doesn’t go down.”*	*“Reflux? Well*,* look—it’s true that I’m better. I’m still having reflux*,* but I’ve noticed that the intensity of the reflux has decreased quite a lot. Quite a lot. I said I didn’t think that much had improved*,* but it’s true*,* now that you talk about the details*,* I do notice it. Yeah*,* that’s true. I have much less reflux.”*
Abdominal pain or stomachache n = 6	Abdominal pain or stomachache n = 1		No changes reported	*“If I have peanuts*,* it feels like I’m having a complete heart attack. It’s just awful … I’ll have more pain in the abdomen region*,* and kind of like essentially where I would physically imagine my sphincter being. That’s kind of where the pain is*,* or if you’re kind of looking at your solar plexus area like that little region there between the breasts or whatever. That’s usually where the pain will be really intense.”*	*“What I have is gas. Maybe it’s swallowing*,* drinking water to push the food. What I notice is a large increase in the gas I have in my stomach. I have no pain. It’s true*,* yes*,* it has been decreased a little bit.”*
Pain in chest n = 4	Pain in chest n = 2		No changes reported	*“For my entire lifetime*,* I haven’t been able to eat honey or food with honey*,* because when I eat food with honey... I feel a very intense pain exactly—or very similar to—when you are impacted*,* as if someone was inserting a burning needle in my pylorous area. The difference is that one is instant [honey]... and with food*,* let’s say fatty food and so on*,* well it’s being digested and it takes a while.”*	*“So*,* Monday I woke up*,* and I was just in a lot—a lot of pain. My esophagus—like it—my chest just hurt really badly. Again*,* I know it’s my esophagus*,* but you always have that brief moment of like*,* ‘Oh my god*,* is it my heart this time?’. It was just*,* yeah*,* a lot of really bad chest pain. It lasted the whole day.”*
Heartburn n = 3	Heartburn n = 1		Less heartburn during the trial	*“I get heartburn when I drink alcohol or eat something very heavy. I mean stuffing myself or eating a lot for dinner*,* for example. I automatically take medicine when I have heartburn. I take something for my stomach. So*,* I don’t know how long it [would last] because I stop it.”*	*-*

For some patients, the slight reduction in symptoms did not lead to significant improvements in the impact of EoE on their daily lives. However, a few patients reported a boost in confidence and an improvement in their overall emotional well-being due to participation in the trial. They became more socially engaged, enjoyed eating, and focused on cultivating a healthier lifestyle, fostering an improved emotional outlook. Increased awareness about living with and managing EoE was a positive outcome. Improvement in swallowing elevated confidence, making eating and socializing more appealing. However, food planning time increased during the trial, and work-related impacts and difficulty selecting restaurants persisted.

### Experience with trial and perspectives on successful treatment

Patients, reflecting on what a successful treatment would mean for them, expressed a range of expectations. One patient conveyed a significant fear regarding the worsening of EoE symptoms and defined treatment success as not having to grapple with that fear any longer. Three patients envisioned treatment success as nothing less than a complete resolution of symptoms, allowing them to eat “without having issues swallowing on a daily basis,” while one patient modestly viewed successful treatment as a reassurance that symptoms would not worsen. Other patients also related successful treatment with the abatement of symptoms, emphasizing the importance of achieving relief from the challenges of swallowing. Patients considered a reduction in symptoms, particularly choking incidents, and the presence of mucus in the throat, as integral components defining a successful treatment.

The trial experience was perceived by most patients (*n* = 9/11) to be positive, as trial participation boosted their confidence and gave them hope, ameliorating the emotional burden associated with the disease. Furthermore, patients raised concerns of not being able to continue treatment with the study drug (due to access) when the trial ended. This underscored the importance patients placed on the benefits derived from trial participation and their desire to maintain the positive trial experience in the ongoing management of EoE.

## Discussion

The patient perceptions gathered from the interviews provide insights into the experience of living with EoE. Patients described their disease symptoms, behavioral adaptations, and repercussions on daily life, as well as their expectations from treatment and perceptions of a successful clinical trial. This qualitative analysis helped us achieve a deeper understanding of the patient experience with EoE and the impacts EoE symptoms have on daily life. Our study highlights the effect that clinical trial participation can have on patients’ confidence, emotional well-being, and disease awareness. This novel insight reveals how trial participation, regardless of treatment efficacy, influences patients’ perceptions of their condition, potentially enabling them to better manage their disease.

In the first interview, the primary concern reported by all patients was difficulty swallowing (including solid and soft foods, and delayed swallowing), which was expected of a clinical trial population with high levels of dysphagia symptoms as an inclusion criterion. However, the challenges with swallowing extended further to discomfort or pain during swallowing, reflux, heartburn, chest pain, abdominal discomfort, and experiencing regurgitation or vomiting when food lodged in the esophagus. These symptoms are aligned with previously reported clinical presentations of EoE [18, 19]. In the first interview, patients also described the consequences of these symptoms, such as concerns about disease progression and adopting certain eating habits, including avoiding specific foods, consuming smaller portions, and refraining from eating in public. Emotional, social, and professional impacts were described, including frustration, embarrassment, anxiety, and limitations in social activities. Work attendance was affected, and patients perceived a disruption in their sense of normalcy, impacting family and romantic relations. Patients expressed varied expectations for a successful treatment, ranging from alleviating fears of symptom worsening to complete resolution of symptoms and relief from swallowing challenges. A reduction in symptoms, especially choking incidents and throat mucus, was considered integral to defining treatment success. These results are consistent with a previous cross-sectional qualitative research study on the journey of patients with EoE and their care partners from the US, that reported physical as well as emotional burden of the disease with limitations in social activities, education, and work [20].

The second interview in this study underlined the evolving experiences of patients with EoE, while the overarching symptom continued to be the challenge with swallowing. Patients conveyed a spectrum of experiences with varying degrees of symptom relief or persistence. The patients who reported improvements in the EoE symptoms were uncertain if such positive changes were due to the study treatment or a shift in their disease management perception stemming from their trial participation. The patients continued to be burdened by emotions such as anxiety, worry, and embarrassment, emphasizing the ongoing challenges faced by individuals with EoE. Despite this, the trial experience was generally positive, boosting confidence and providing hope, though concerns arose about discontinuing the study drug post-trial. Patients expressed a desire to maintain the positive trial experience in ongoing EoE management. They described the impact of EoE symptoms on various facets of their daily lives, mostly focusing on eating habits. Adopting strategies such as avoiding specific foods, consuming smaller portions, and refraining from eating in public were recurrent lifestyle modifications. A complex interplay between symptom improvement and dietary modifications was noted, highlighting the importance of personalized dietary adjustments in managing EoE symptoms as has previously been reported [3, 13].

The MESSINA clinical trial did not show a statistically significant effect on symptom reduction, and it should be noted that the minor symptom improvements reported were individual cases and not representative of a broader trend. While treatment did not directly lead to significant symptom relief, trial participation encouraged a more active engagement with disease management, which resulted in improved confidence and a more positive outlook on daily activities, such as eating and socializing.

Overall, the implications of EoE extended beyond the physical challenges, permeating into emotional, social, and professional domains. Such disease challenges have been seen to negatively affect patients’ quality of life [9, 16]. Patients felt they were unable to participate in activities they considered ‘normal’, such as sports and traveling, which affected their emotional well-being. A recent qualitative research study on adult patients with EoE from eight different countries highlighted a lack of physicians’ and patients’ awareness of the emotional burden of the disease, which adversely impacted the patient journey [20]. The researchers revealed the importance of acknowledging the emotional burden of the disease, particularly during the initial stages where the patient-reported symptoms were often overlooked due to lack of disease awareness, empathy, and proactivity from physicians [21]. During the monitoring stage of disease management in the study (i.e., post-treatment and follow-up), patients presented with positive emotions of comfort, happiness, and control, consistent with the results of the current study [21].

There are certain limitations, beyond the small sample size, that need to be considered when interpreting these findings. The second interview focused on change during the trial, with regards to both the symptoms and the impact of symptoms on daily life. However, as the two interviews were almost a year apart, there could be challenges with data that were based on a recall over an extended period. The absence of a follow-up third interview restricted the possibility to track long-term impacts on patients’ lives and the evolving experience of patients over time. A detailed longitudinal analysis was not possible due to the study design, and therefore time-related differences were not explored. As the MESSINA inclusion criteria required patients to have moderate-to-severely symptomatic EoE, and the interview sub-study only recruited adults, the results of this study may not be applicable to patients with milder disease or to children or adolescents. It is pertinent to highlight that the positive impacts on confidence and social engagement reported by patients may be influenced by the increased attention they received during the trial process, rather than the treatment itself. This limitation should be considered when interpreting relevant findings. Despite these limitations, this study offers an in-depth and comprehensive exploration of patient experiences with EoE and underscores the importance of incorporating patient perspectives into clinical decision making to improve care for individuals with EoE. These insights on patients’ increased confidence and disease awareness during the trial show the importance of integrating patient education and emotional support in clinical research and standard care to help patients manage their disease.

## Conclusion

This qualitative in-trial, patient interview study described patient perceptions of EoE symptoms, focusing on how these affected their daily lives. Difficulty swallowing, pain, choking, and the fear of choking, as well as behavioral adaptations were affecting patients emotionally and socially. The encouraging responses from patients regarding their participation in the trial highlight the importance of capturing patient insights during a trial to facilitate the development of treatments that address patients’ needs and concerns.

## Electronic supplementary material

Below is the link to the electronic supplementary material.

Supplementary Material 1

## Data Availability

The authors confirm that the data supporting the findings of this study are available within the article and its supplementary materials. Sharing further information on the patient transcripts or coded quotes may present a risk for patient identification.
