# Supplementary material for: Patient experience with eosinophilic esophagitis symptoms and impacts on daily life based on in-trial qualitative interviews
Source: J Patient Rep Outcomes. 2025 Jan 8;9:3. doi: 10.1186/s41687-025-00836-x (PMC11711566; doi:10.1186/s41687-025-00836-x)
Supplement: Supplementary file 1 — Supplementary Material 1 [file 41687_2025_836_MOESM1_ESM.docx]

# **SUPPLEMENTARY MATERIAL**

**Supplementary Table 1: Demographics for all patients who completed the first interview**

| Demographics | N=34 |
| --- | --- |
| **Age (in years)** |  |
| Mean (SD) | 41 (11.6) |
| Range | 18–67 |
| Not reported | 1 |
| **Age at diagnosis** |  |
| Mean (SD) | 34 (11.1) |
| Range | 16–64 |
| Not reported | 2 |
| **Time since diagnosis ranges** |  |
| Less than 1 year | 3 (8.8%) |
| 1–4 years | 8 (23.5%) |
| 5–9 years | 4 (11.8%) |
| 10–14 years | 9 (26.5%) |
| 15 years or more | 5 (14.7%) |
| Not reported | 5 (14.7%) |

SD: standard deviation.
